# Striatin heterozygous mice are more sensitive to aldosterone-induced injury

**DOI:** 10.1530/JOE-19-0562

**Published:** 2020-03-31

**Authors:** Amanda E Garza, Elijah Trefts, Isis A Katayama Rangel, Danielle Brooks, Rene Baudrand, Burhanuddin Moize, Jose R Romero, Sanjay Ranjit, Thitinan Treesaranuwattana, Tham M Yao, Gail K Adler, Luminita H Pojoga, Gordon H Williams

**Affiliations:** 1 Division of Endocrinology, Diabetes and Hypertension, Department of Medicine, Brigham and Women’s Hospital, Harvard Medical School, Boston, Massachusetts, USA; 2 Department of Endocrinology, School of Medicine, Pontificia Universidad Catolica De Chile, Santiago, Chile

**Keywords:** mineralocorticoid receptor antagonist, aldosterone, cardiac and renal injury, non-genomic, striatin

## Abstract

Aldosterone modulates the activity of both epithelial (specifically renal) and non-epithelial cells. Binding to the mineralocorticoid receptor (MR), activates two pathways: the classical genomic and the rapidly activated non-genomic that is substantially modulated by the level of striatin. We hypothesized that disruption of MR’s non-genomic pathway would alter aldosterone-induced cardiovascular/renal damage. To test this hypothesis, wild type (WT) and striatin heterozygous knockout (Strn^+/^^−^) littermate male mice were fed a liberal sodium (1.6% Na^+^) diet and randomized to either protocol one: 3 weeks of treatment with either vehicle or aldosterone plus/minus MR antagonists, eplerenone or esaxerenone or protocol two: 2 weeks of treatment with either vehicle or L-NAME/AngII plus/minus MR antagonists, spironolactone or esaxerenone. Compared to the WT mice, basally, the Strn^+/^^−^ mice had greater (~26%) estimated renal glomeruli volume and reduced non-genomic second messenger signaling (pAkt/Akt ratio) in kidney tissue. In response to active treatment, the striatin-associated-cardiovascular/renal damage was limited to volume effects induced by aldosterone infusion: significantly increased blood pressure (BP) and albuminuria. In contrast, with aldosterone or L-NAME/AngII treatment, striatin deficiency did not modify aldosterone-mediated damage: in the heart and kidney, macrophage infiltration, and increases in aldosterone-induced biomarkers of injury. All changes were near-normalized following MR blockade with spironolactone or esaxerenone, except increased BP in the L-NAME/AngII model. In conclusion, the loss of striatin amplified aldosterone-induced damage suggesting that aldosterone’s non-genomic pathway is protective but only related to effects likely mediated via epithelial, but not non-epithelial cells.

## Introduction

During the past two decades, a substantial expansion has occurred in understanding aldosterone’s mechanism of action, specifically in two areas – cells targeted and pathways used. First, the tissues that are aldosterone’s targets have expanded beyond its classical modulation of the activity of epithelial (particularly renal) cells, modifying sodium absorption and thereby participating in volume homeostasis, to include actions on non-epithelial cells, for example, cardiac, smooth muscle, endothelial, immune, neural, adrenal. Second, recent studies have documented that when aldosterone binds to the mineralocorticoid receptor (MR), not one, but two, pathways are activated: the classical genomic pathway, where aldosterone/MR acts as a nuclear transcription factor, and the rapidly activated, non-genomic pathway (Fuller & Young 2005, Baudrand *et al.* 2014). Activation of the non-genomic pathway is associated with increased phosphorylation of several proteins, including those in the mitogen-activated protein kinase (MAPK) pathway and with intracellular calcium regulation (Funder 2017).

It is well known that activation of the aldosterone/MR pathway, in the presence of a liberal sodium (1.6% Na^+^), diet, is a risk factor for a variety of cardiovascular/renal diseases and that MR antagonists (MRA) can ameliorate these adverse effects (Takeda *et al.* 2000, Martinez *et al.* 2002, Rocha *et al.* 2002, Endemann *et al.* 2004, Jain *et al.* 2009). However, it is unclear whether the aldosterone-induced cardiovascular and renal damage is being mediated by its actions on epithelial (renal, volume) and/or non-epithelial cell types. In addition, it is uncertain what role, if any, the MR non-genomic pathway plays in mediating these adverse events.

We have previously documented that striatin, a scaffold, and signaling protein, modulates the activity of aldosterone/MR’s non-genomic pathway, *in vivo* and *in vitro* (Pojoga *et al.* 2012, Garza *et al.* 2015*a*,*b*). In cultured human endothelial cells (EA.hy926, human umbilical vein cell line), knocking down striatin prevents the activation of the non-genomic, but not the genomic pathways (Coutinho *et al.* 2014). On a liberal sodium diet (LibS, 1.6% Na^+^), striatin heterozygote-knockout mice (Strn^+/^^−^) exhibit increased MR genomic activity but blunted MR non-genomic activity. These mice have salt-sensitivity of blood pressure (BP), enhanced vascular contractility, and increased plasma aldosterone compared to wild type littermates (Garza *et al.* 2015*a*,*b*). Further, in humans, we identified an association between single nucleotide polymorphic (SNP) variants of the striatin gene and salt-sensitive BP in hypertensive subjects (Garza *et al.* 2015*b*). Finally, we have documented that aldosterone and the level of sodium intake can modulate striatin levels (Pojoga *et al.* 2012).

Among the established experimental models of cardiovascular and renal damage, two involving MR activation are illustrative of contrasting approaches – exogenously administered aldosterone and NG-nitro-L-arginine methyl ester (L-NAME) induced deficiency of nitric oxide/angiotensin II (AngII) (Oestreicher *et al.* 2003). Both require a liberal sodium diet (1.6% Na^+^) to produce the damage. Injury in both models is MR mediated as treatment with an MRA prevents the damage. While BP is increased in both models, hypertension (HTN) does not appear to be a critical mediator of the damage, as MR antagonism reduces the damage without having much if any effect on BP at least in the L-NAME/AngII model (Oestreicher *et al.* 2003). Yet the aldosterone levels are different – high in the exogenous aldosterone and low in the L-NAME/AngII model. The damage in the former model is presumed largely to be secondary to volume expansion via activation of MR on epithelial cells. The damage in the L-NAME/AngII model may largely be secondary to the activation of MR on non-epithelial cells. However, whether the damage in either model is mediated by MR’s genomic and/or non-genomic pathways is unknown.

Using a mouse model of reduced striatin expression, Strn^+/^^−^, and thereby reduced non-genomic pathway activity, we assessed the hypothesis that MR’s non-genomic signaling pathway modifies aldosterone-induced cardiovascular and renal damage. Based on our and others, previous studies, we predict that cardiac and renal damage would increase when striatin is reduced. However, it is uncertain whether the effect of striatin genomic reduction would be the same in both models. To assess the specificity of our findings were related to activation of the MR, we used two MRA’s – eplerenone (steroid based) and esaxerenone (non-steroid based).

## Materials and methods

### Study protocols

Procedures were approved by the Institutional Animal Care and Use Committee at Brigham and Women’s Hospital. Twelve to 16-week-old male WT and Strn^+/−^ male mice were acclimatized to BP measurements for 1 week. The mice were randomized to one of two protocols and fed a liberal sodium (LibS, 1.6% Na^+^) (Test Diet) diet for the duration of the study. Tail-cuff BP was assessed at baseline and the end of the study, as previously described (Garza *et al.* 2015b).

#### Aldosterone protocol

Mice from each genotype were randomized to receive one of the following treatments (8 mice/group) for 3 weeks: (1) control (vehicle), (2) aldosterone 200 µg/kg/day, (3) aldosterone plus 100 mg/kg/day eplerenone; or (4) aldosterone plus 1 mg/kg/day esaxerenone. Aldosterone (Sigma) was delivered via s.c. Alzet osmotic minipumps (Model 1004). Eplerenone and esaxerenone (Daiichi Sankyo, Tokyo, Japan) were delivered in the LibS food. On day 19, BP was measured, and mice were placed in metabolic cages for 24 h for the collection of urine. On day 22, blood was collected by submandibular vein sampling, and mice were euthanized under deep anesthesia with isoflurane, heart and kidneys excised, tissue flash-frozen, and stored at −70°C or fixed for histological analysis.

#### L-NAME (LN)/Angiotensin II (AngII) protocol

Mice from each genotype were randomized: (1) control (vehicle), (2) L-NAME plus AngII (Sigma) (LN/AngII), (3) LN/AngII plus spironolactone (100 mg/kg/day) or (4) LN/AngII plus esaxerenone (3 mg/kg/day). L-NAME was administered in the drinking water 0.2 mg/mL/day. On day 7, L-NAME-treated mice received AngII 0.7 mg/kg/day, via Alzet osmotic minipumps (Model 1007D) for the remainder of the study. On days 10 and 13, the same procedures were performed as noted above for day 19 and 22 in the aldosterone protocol.

### Blood pressure measurement

BP was measured using the CODA noninvasive BP system (Kent Scientific), as previously described (Pojoga *et al.* 2011). Briefly, mice were kept warm at 37°C for 5 min allowed to rest quietly, and 35 cycles were measured and analyzed using Kent scientific software. The same individual handled mice for the entire study.

### Urine and plasma analysis

On day 19 of the aldosterone protocol or day 10 of the L-NAME/AngII protocol, mice were individually housed in metabolic cages for measurement of 24-h water and food intake and collection of 24-h urine. Urine was stored at −20°C until analyzed. Twenty-four-hour proteinuria was assessed using the microalbumin/creatine DCA 2000 Reagent kit (Siemens). Blood was collected in microtainer tubes with EDTA (BD Microtainer), and plasma was collected following centrifugation. Aldosterone levels were determined using a solid-phase RIA kit (Siemens Diagnostic Products) using duplicate samples as previously described (Garza *et al.* 2015*b*). To measure urine aldosterone excretion, a 24-h urine sample was subjected to acid hydrolysis and extraction. Plasma renin active (PRA) was measured by RIA, according to the manufactures instructions and as previously described (DiaSorin, Minneapolis, MN, USA) (Garza *et al.* 2015*b*).

### Histology

Kidney and heart tissues were excised, rinsed in PBS, and immediately immersed in 10% formalin/4% formaldehyde for 24 h. Tissues were subsequently placed in 70% ethanol until paraffin embedding, sectioning, and staining. Both heart and kidney tissues were stained with hematoxylin and eosin (H&E). Kidney tissue was stained with periodic acid-Schiff (PAS). Processing and staining were performed by the Histopathology Rodent Core, Harvard Medical School. Estimated glomerular volume was measured as previously described (Guo *et al.* 2006). Myocardial injury was determined using heart sections stained with hematoxylin and eosin (H&E). These sections were examined by light microscopy and scored on a scale of 0–4 where: 0, represents no damage; 1, represents isolated damage focal interstitial inflammatory infiltrates without myocyte injury, 2, represents one focal area of damage of interstitial inflammatory infiltrate associated with myocyte injury; 3, two or more areas of damage with inflammatory infiltrates; and 4, diffuse areas of damage in more than 50% of the myocardium with inflammatory infiltrates.

### Nanostring technologies

RNA was isolated from flash frozen tissue using an RNeasy Kit (Qiagen), and 100 ng of RNA was processed by the Molecular Biology Core at Dana-Farber Cancer Center, Boston, MA, USA. Samples were assessed for quality and concentration (Agilent Bioanalyzer RNA Nano or Pico chips); smear analyzed (Agilent 2100 Expert software) to quantify the percentage of RNA fragments greater than 300 nt; capture and reporter Code sets added; samples hybridize at 65°C for 16 hours, washed and loaded onto a custom-made cartridge (XT-GXA-P1CS-096) using the nCounter Analysis System Prep Station. The cartridge was scanned using the nCounter Digital Analyzer at the maximum resolution of 1150 FOV. Data analysis was completed using nSolver software. Gene expression normalization was performed using the housekeeper genes, *tbp* and *tubb5*.

### Statistics

Data are presented as mean ± s.e unless otherwise noted and were analyzed using an ANOVA with *post hoc* analysis as indicated. Student’s *t*-test or nonparametric tests were performed for unpaired data for comparison of two means. The following designations were used to identify the degree of significance: ns = *P* > *0.05*, * = *P* < 0.05, ** = *P* < 0.01, ***P < 0.001.

## Results

### Aldosterone protocol

#### Striatin deficiency significantly increased salt sensitivity of blood pressure

**Figure 1:**
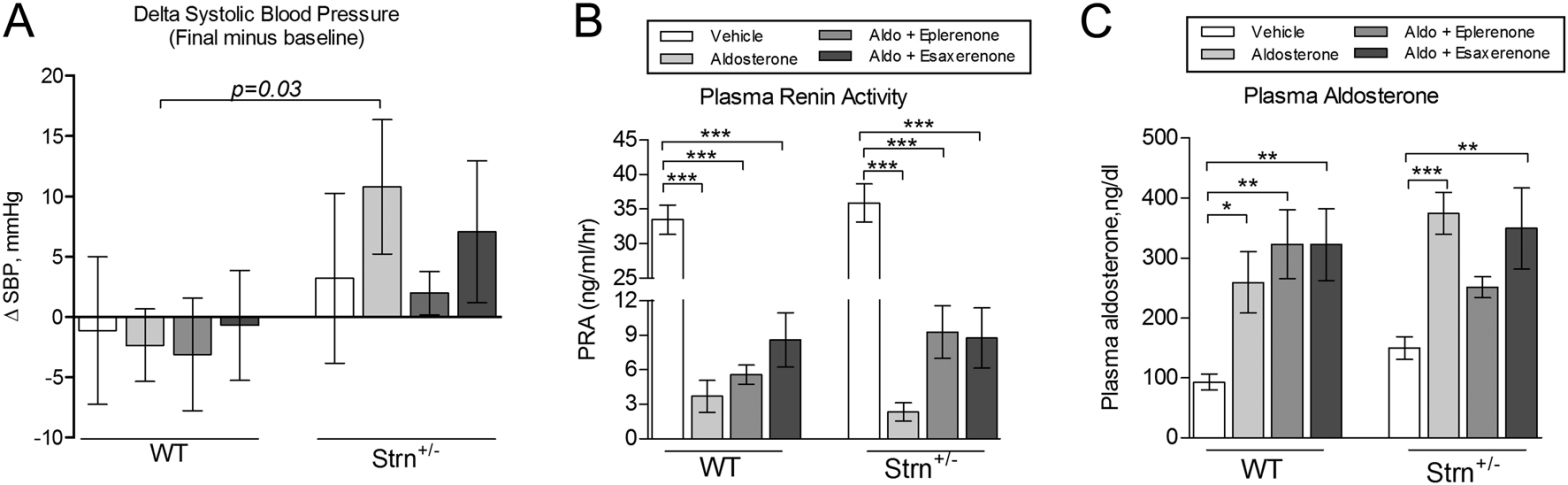
Blood pressure and activation of RAAS after 3 weeks of treatment. (A) Delta systolic BP measured as treatment BP minus baseline BP, *n* = 7–8/group. Data represent mean ± s.e.m. (B) Plasma renin activity (PRA) and (C) plasma aldosterone were measured by radioimmunoassay. Statistical analysis performed using one-way ANOVA with a *post hoc* Bonferroni’s multiple comparison test, * = *P* < 0.05, ** = *P* < 0.01,*** = *P* < 0.001 compared to vehicle and *n* = 5–8/treatment group.

**Figure 2:**
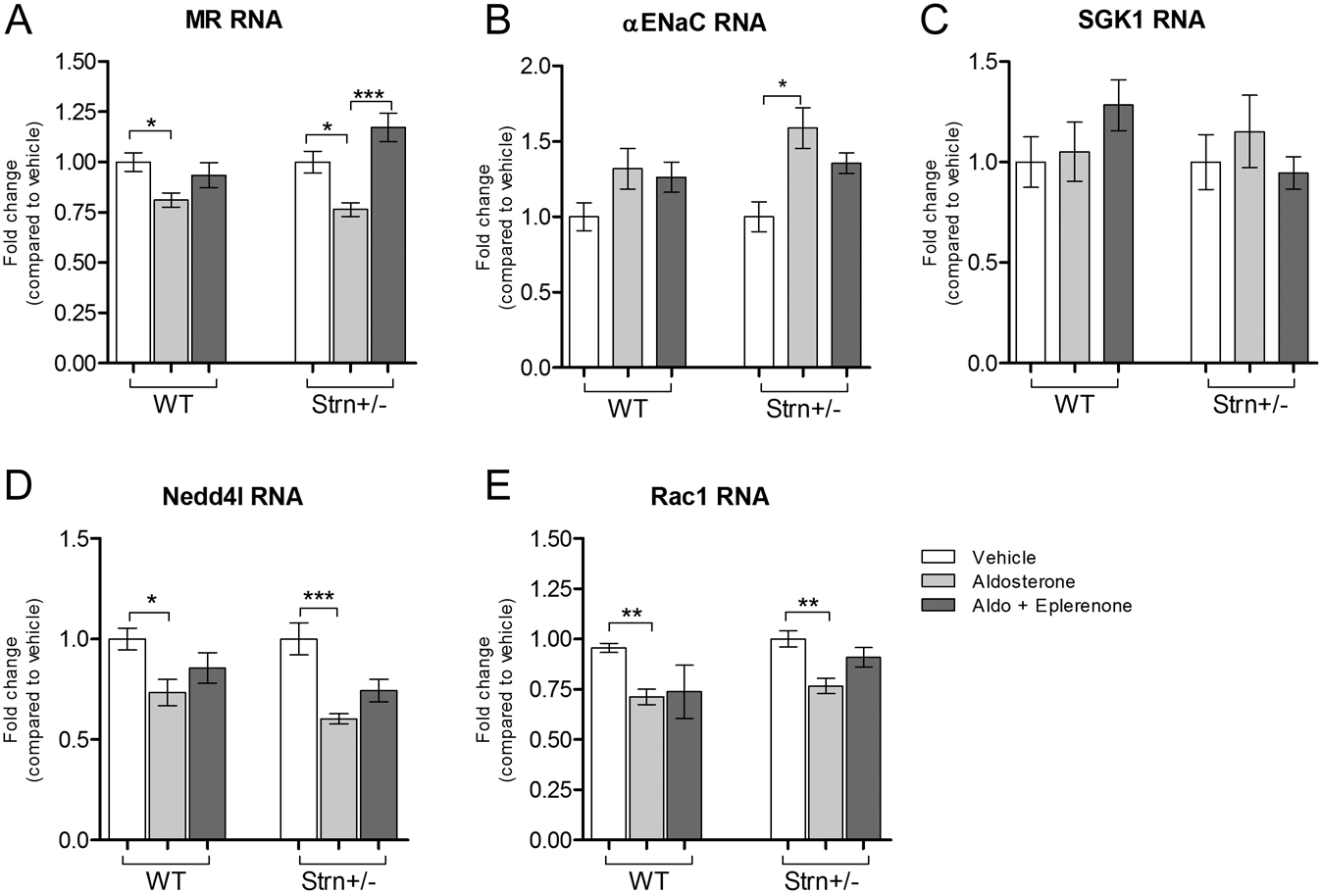
MR genomic markers of renal sodium signaling. (A) MR, (B), αENaC, (C) SGK1, (D) Nedd4l and (E) Rac1 as measured by Nanostring Technologies. *n* = 5–6. Data presented at mean ± s.e.m. ns = *P* > 0.05, * = *P* < 0.05, ** = *P* < 0.01, ***P < 0.001.

Baseline systolic and diastolic BP was not significantly different comparing WT and Strn^+/–^ mice (SBP 103 ± 2 and 99 ± 1, *P* = *0.08* and DBP 74 ± 2 and 71 ± 2 mmHg; *n* = 32/group *P* = *0.21*). After 3 weeks of treatment with aldosterone, WT mice did not exhibit any SBP changes from baseline in any of the four treatment groups (Fig. 1A). In contrast, compared to WT mice, Strn^+/–^ mice had significantly greater salt-sensitivity (five-fold increase) of SBP in response to aldosterone (Fig. 1A). Eplerenone blunted the increase to levels that were similar to the vehicle control group. Esaxerenone did not completely prevent the increases in BP. These differences in salt-sensitive SBP were not associated with significant differences in the expression of commonly associated aldosterone-mediated genomic biomarkers: MR, αENaC, SGK1, Nedd4l, and Rac1 (Fig. 2A, B, C, D and E). These data suggest that with aldosterone infusion, a reduction in striatin protein is associated with an increase in SBP. However, this response is not secondary to aldosterone’s genomic mechanism of actions as molecular markers of this mechanism did not differ by genotype.

#### Striatin deficiency significantly increased renal damage associated with aldosterone infusion

**Figure 3:**
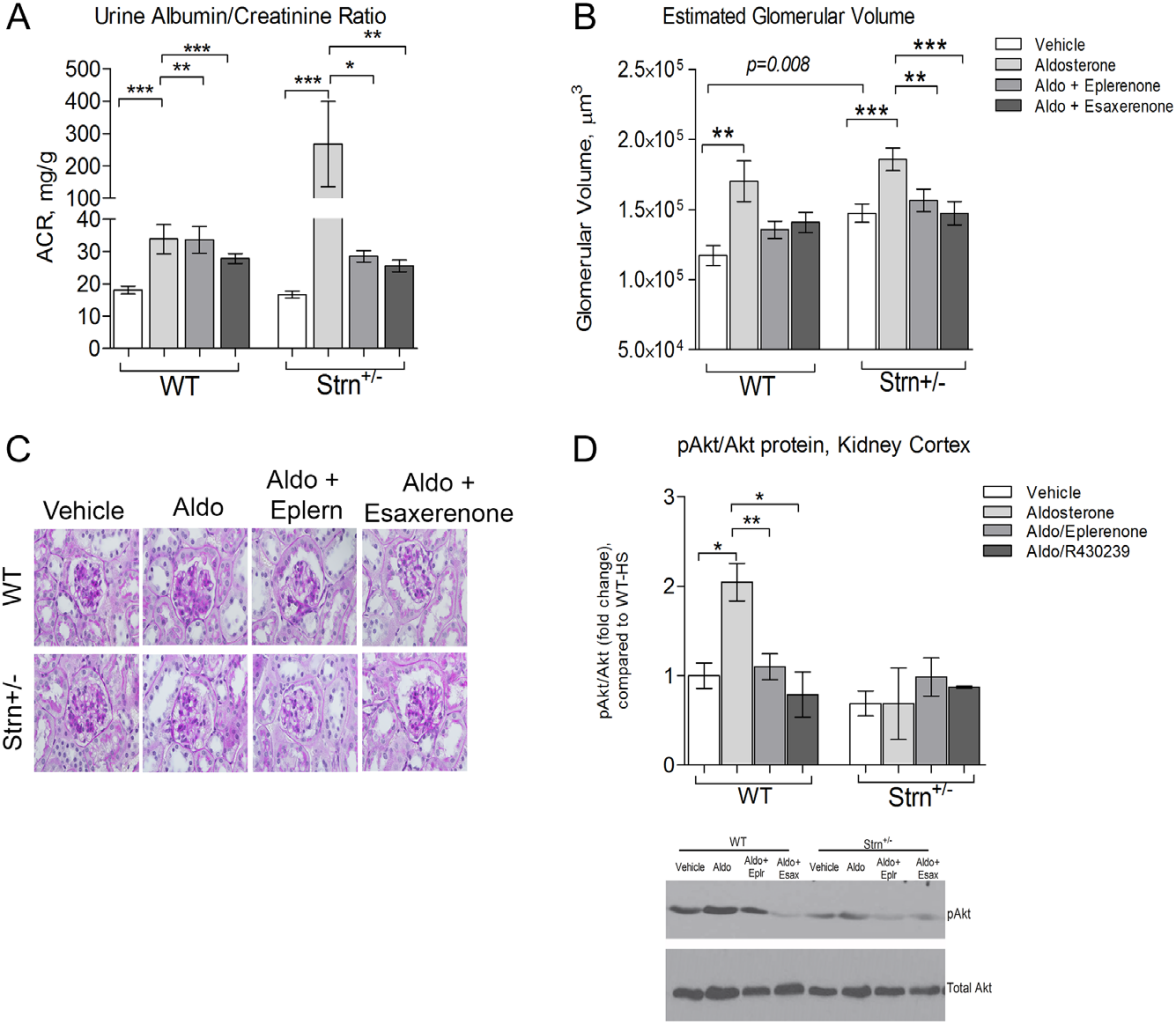
Renal injury. (A) Urine albumin/creatinine ratio was measured using DCA systems microalbumin/creatinine regent kit. (B) Estimated glomerular volume was performed by Periodic acid-Schiff (PAS). (C) Representative glomeruli section, magnification 600×. Data presented as mean ± s.e.m., *n* = 6–8 mice/group. (D) pAkt/Akt renal cortex protein analysis by western immunoblot. Data presented as mean ± s.e.m., *n* = 6–8 mice/group. Statistical analysis, * = *P* < 0.05, ** = *P* < 0.01, *** = *P* < 0.001 one-way ANOVA analysis comparing treatment groups within each genotype.

Aldosterone infusion caused a significant increase in microalbuminuria in both WT and Strn^+/−^ mice. However, the increase observed in the Strn^+/−^ mice was significantly greater (*P* = *0.0022*) than in the WT mice (Fig. 3A) with MR blockade substantially reducing the microalbuminuria levels. To assess an additional measurement of kidney injury, we quantified glomerular volume (GV) in periodic acid-Schiff (PAS) stained kidney slices (Fig. 3B and C). Strn^+/^^−^ mice in the vehicle treatment group had significantly larger GV than WT (1.6 × 10^5^ ± 1.6 × 10^4^ vs 1.9 × 10^5^ ± 4.6 × 10^3^ µm, *P* = *0.008*). Both WT and Strn^+/−^ mice in the aldosterone treatment groups had significantly increased GV as compared to their vehicle groups (Fig. 3B), but this increase did not differ between the two mice groups. Further, the increase in GV was reduced by both MRAs.

Both the kidney weight and kidney weight to body weight ratio (KW/BW) in WT and Strn^+/^^−^ mice increased with aldosterone administration when compared to their respective vehicle treatment group with MR blockade normalizing these changes (Table 1).

**Table 1:** Heart and kidney weight.

	Vehicle	Aldo	Aldo + Eplerenone	Aldo + Esaxerenone
Body weight g, BW				
WT	28 ± 1	28 ± 1	28 ± 1	28 ± 1
Strn^+/-^	28 ± 1	27 ± 1	28 ± 1	27 ± 1
Heart, HW mg				
WT	130 ± 5	140 ± 3	141 ± 7	138 ± 4
Strn^+/-^	129 ± 4	127 ± 10	133 ± 4	138 ± 5
HW/BW mg/g				
WT	4.7 ± 0.3	5.0 ± 0.1	5.0 ± 0.3	4.9 ± 0.1
Strn^+/-^	4.7 ± 0.2	4.7 ± 0.7	4.8 ± 0.2	5.1 ± 0.2
Kidney, KW mg				
WT	189 ± 9	215 ± 8^a^	208 ± 3	199 ± 3
Strn^+/-^	189 ± 7	210 ± 5^a^	199 ± 5	192 ± 4
KW/BW mg/g				
WT	6.8 ± 0.2	7.7 ± 0.2^b^	7.4 ± 0.2	7.1 ± 0.2
Strn^+/-^	6.9 ± 0.2	7.8 ± 0.3^b^	7.2 ± 0.2^b^	7.1 ± 0.1

Data represent mean ± s.e., *n* = 6–8/group. One-way ANOVA with* post hoc* Bonferroni’s statistical analysis performed, vehicle vs aldosterone; aldosterone vs eplerenone and aldosterone vs R430239. ^a^*P*< 0.05; ^b^*P*< 0.01.

In renal cortex tissue, phosphorylation of Akt to total Akt ratio was significantly increased by aldosterone infusion in WT mice, but as anticipated, not in the Strn^+/−^ mice (Fig. 3D).

#### Striatin deficiency did not alter renal biomarkers to aldosterone infusion

**Figure 4:**
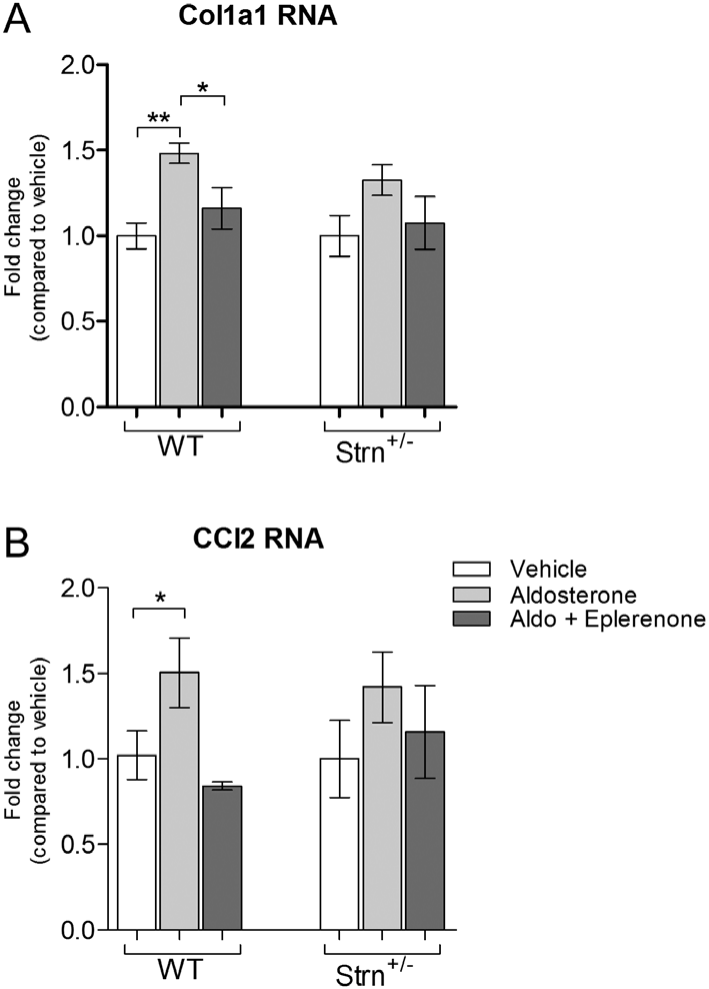
Aldosterone-induced biomarkers of damage. (A) Collagen type I alpha 1 (Col1a1), (B) C-C motif chemokine ligand 2 (CCL2). As measured by Nanostring Technologies. *n* = 5–6. Data presented at mean ± s.e.m. ns = *P* > 0.05, * = *P* < 0.05, ** = *P* < 0.01, ***P < 0.001.

Non-classical aldosterone actions activate various pathways associated with the pathophysiology of aldosterone-induced damage. The expression of collagen type I alpha 1 (Col1a1) and cytokine C-C motif chemokine ligand2 (CCL2), two biomarkers of tissue damage, were upregulated in aldosterone treated WT and Strn^+/−^ mice (Fig. 4A and B). Eplerenone blunted the increase in expression and Strn^+/−^ genotype did not affect this blockade.

#### Striatin deficiency did not alter the cardiac responses to aldosterone infusion

**Figure 5:**
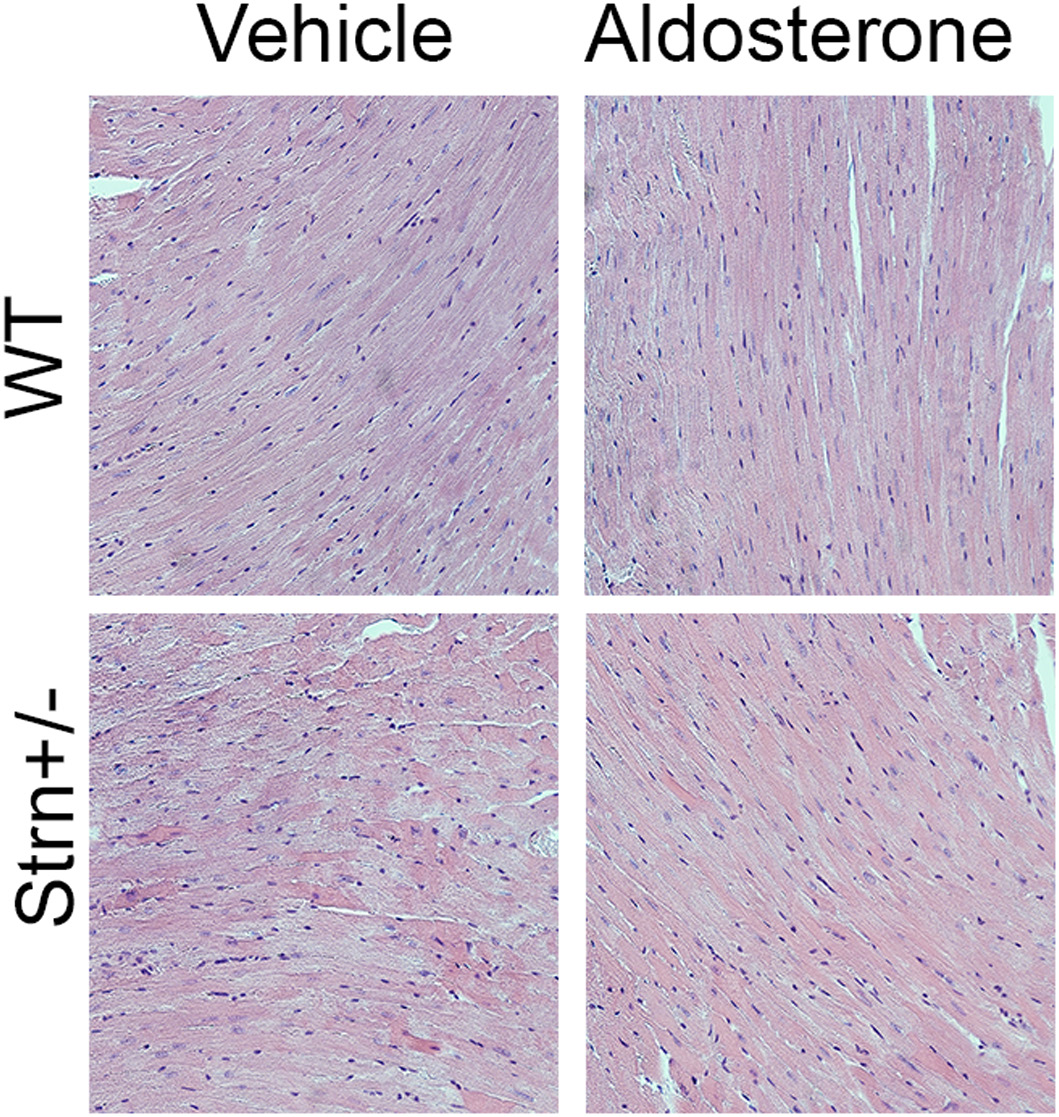
Cardiac tissue analysis myocardial damage, H&E staining, magnification at 200×.

There were no significant differences in heart weight or heart weight/body weight ratio in any of the four treatment groups or two genotypes (Table 1). As a measure of cardiac injury, as described in the methods section. Macrophage infiltrates and focal adhesions were minimal to not observed in the aldosterone-treated cardiac tissue of both WT and Strn^+/–^ mice (Fig. 5).

#### Striatin deficiency did not alter the response of the renin-angiotensin-aldosterone system (RAAS) to aldosterone infusion

The physiological consequence of a high sodium diet in the presence of exogenous aldosterone administration is to suppress the activity of the endogenous renin-angiotensin-aldosterone system (RAAS), as reflected in a reduction in plasma renin activity (PRA) levels. This volume feedback system response was not modified by a reduction in striatin levels as the PRA responses, both to the aldosterone infusion (suppression) and MR blockade (increase), were similar in WT and Strn^+/–^ mice (Fig. 1B). As previously reported (Garza *et al.* 2015*a*,*b*), the Strn^+/–^ mice have significantly higher levels of aldosterone on a high sodium diet compared to WT mice (93 ± 13 vs 150 ± 19, *P* = *0.03*) (Fig. 1C). However, the levels achieved with aldosterone infusion and MR blockade in the two genetic groups were similar. These data suggest that reduction of striatin levels does not modify either the aldosterone or PRA levels in response to chronically administered aldosterone in mice on a high salt diet.

#### Striatin deficiency did not induce changes in weight or food and water intake in response to aldosterone infusion

Mice of both genotypes and in all the treatment groups thrived throughout the experimental period, consuming similar quantities of food (Table 2) and having similar body weights (Table 1). Both WT and Strn^+/–^ aldosterone infused mice drank significantly more water compared to vehicle-treated mice. MR antagonism, either eplerenone or esaxerenone, reduced water intake compared to aldosterone; however, water intake remained higher than the vehicle treatment group (Table 2). This increase in water consumption was associated with increased urine production.

**Table 2:** Metabolic cage.

	Vehicle	Aldo	Aldo + Eplerenone	Aldo + Esaxerenone
Food, mg/day/g, BW				
WT	108 ± 10	96 ± 15	112 ± 13	121 ± 20
Strn^+/-^	117 ± 11	120 ± 15	106 ± 10	105 ± 16
Water, µL/day/g, BW				
WT	156 ± 25	331 ± 47^b^	220 ± 30	282 ± 36
Strn^+/-^	150 ± 20	439 ± 37^c^	268 ± 43^a^	270 ± 50^a^
Urine, µL/day/g, BW				
WT	65 ± 5	195 ± 26^c^	104 ± 12^c^	140 ± 14
Strn^+/-^	67 ± 11	194 ± 22^c^	127 ± 7^c^	127 ± 18^a^

Data represent mean ± s.e., *n* = 7–8/group. One-way ANOVA with* post hoc* Bonferroni’s statistical analysis performed: vehicle vs aldosterone and aldosterone vs MRA. ^a^*P* < 0.05; ^b^*P* < 0.01; ^c^*P* < 0.001.

### L-NAME/AngII protocol

#### Striatin deficiency did not modify L-NAME/AngII induced changes in blood pressure

**Figure 6:**
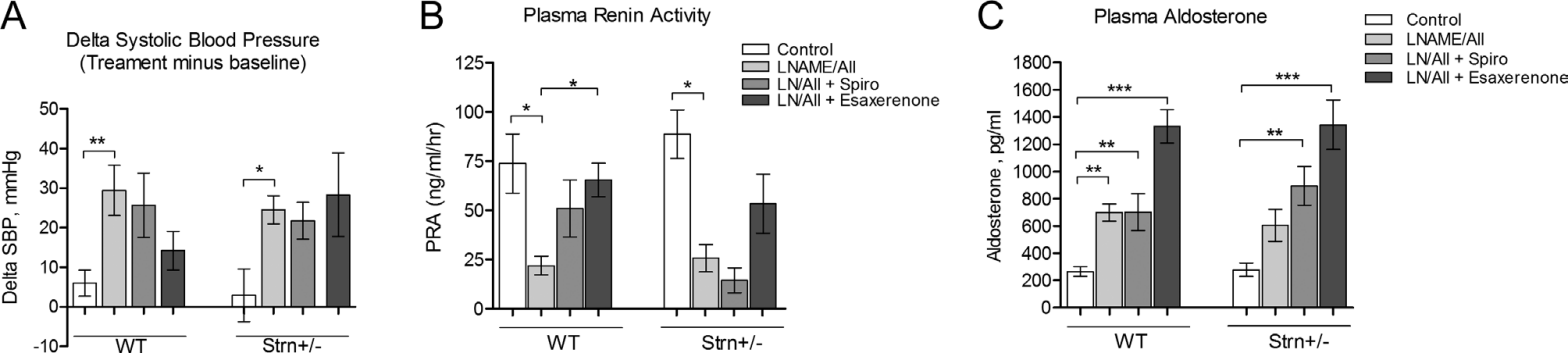
The effect of L-NAME/AngII treatment on blood pressure and RAAS. (A) Delta SBP was measured as a change from day 11 minus day baseline. (B) Plasma aldosterone and (C) plasma renin activity (PRA) were measured by radioimmunoassay. Statistical analysis performed using one-way ANOVA with a post hoc Bonferroni’s multiple comparison test, * = *P* < 0.05, ** = *P* < 0.01, *** = *P* < 0.001 compared to vehicle and *n* = 5–8/treatment group. One-way ANOVA with Bonferroni *post hoc* analysis compared to the control group within each genotype *P < 0.05, **P < 0.01, ***P < 0.001; *n* = 6–13.

BP was measured at baseline, prior to randomization and was similar between WT and Strn^+/^^–^ groups (111 ± 2 and 112  ± 2 mmHg, respectively). On study day 10, systolic BP was significantly higher in both WT and Strn^+/–^ mice treated with L-NAME/AngII compared to the control groups (Fig. 6A). Systolic BP in L-NAME/AngII treated was not significantly different between WT and Strn^+/–^ mice. In the WT mice, MR blockade with spironolactone did not prevent the rise in BP in L-NAME/AngII-treated mice (Fig. 6A). We have previously observed similar results in rats (Oestreicher *et al.* 2003). The non-steroidal MR antagonist, esaxerenone did not completely blunt the rise in systolic BP in response to L-NAME/AngII treatment; however, BP was no longer significantly different from the control group (Fig. 6A). In the Strn^+/–^ group, neither MR antagonist, spironolactone nor esaxerenone, was able to blunt the rise in BP; however, there was a decreasing trend. A reduction of striatin protein prevents the ability of esaxerenone to effectively blunt the L-NAME/AngII induced increases in systolic BP.

At baseline, total body weight was not different between genotypes or treatment groups (WT, 29 ± 0.4 and Strn^+/^^–^ 29 ± 0.7 g, *P* = 0.72. At the conclusion of the experiment, whole body weight was significantly reduced in the L-NAME/AngII treated WT mice compared to WT control mice.

#### Striatin deficiency did not modify L-NAME/AngII induced cardiac injury

**Figure 7:**
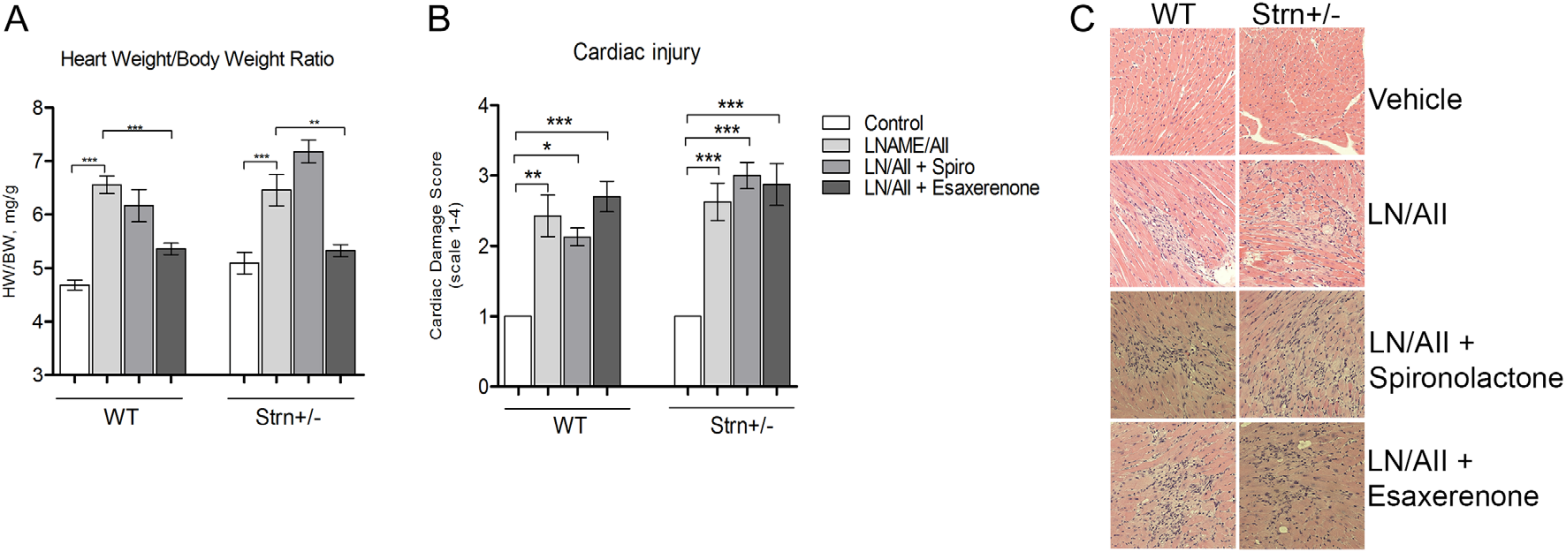
Cardiac injury assessment following L-NAME/AngII treatment. (A) Heart weight (HW) to body weight (BW) ratio was measured after 11 days of treatment. (B) Cardiac damage score using a scale from 1 to 4. (C) Representative myocardial macrophage infiltration and necrotic lesions, hematoxylin and eosin; magnification, ×20. One-way ANOVA, Bonferroni multiple comparison post-hoc to control group within each genotype, *P < 0.05, **P < 0.01 and ***P < 0.001; *n* = 4–10.

As an assessment of cardiac hypertrophy, we measured the heart to body weight (BW) ratio. L-NAME/AngII treatment significantly increases heart weight and the heart/BW ratio (Fig. 7A) to a similar extent in the WT and Strn^+/–^ mice. Spironolactone did not prevent this increase in either WT or Strn^+/–^ mice. In the Strn^+/–^ mice, esaxerenone treatment significantly reduced the cardiac hypertrophy to levels indistinguishable from the placebo group (Fig. 7A). Furthermore, the effect of esaxerenone on Strn^+/–^ mice heart/BW ratio was significantly different from that of spironolactone (6.3 ± 0.3 vs 5.1 ± 0.2, *P* < 0.001). L-NAME/AngII treatment induces significant myocardial injury (Fig. 7B and C). MRA treatment, esaxerenone nor spironolactone could prevent or reduce the number of foci. In the L-NAME/AngII treatment groups, WT mice had a similar injury score to Strn^+/–^ mice.

#### Striatin deficiency did not modify L-NAME/AngII-*induced renal injury*

**Figure 8:**
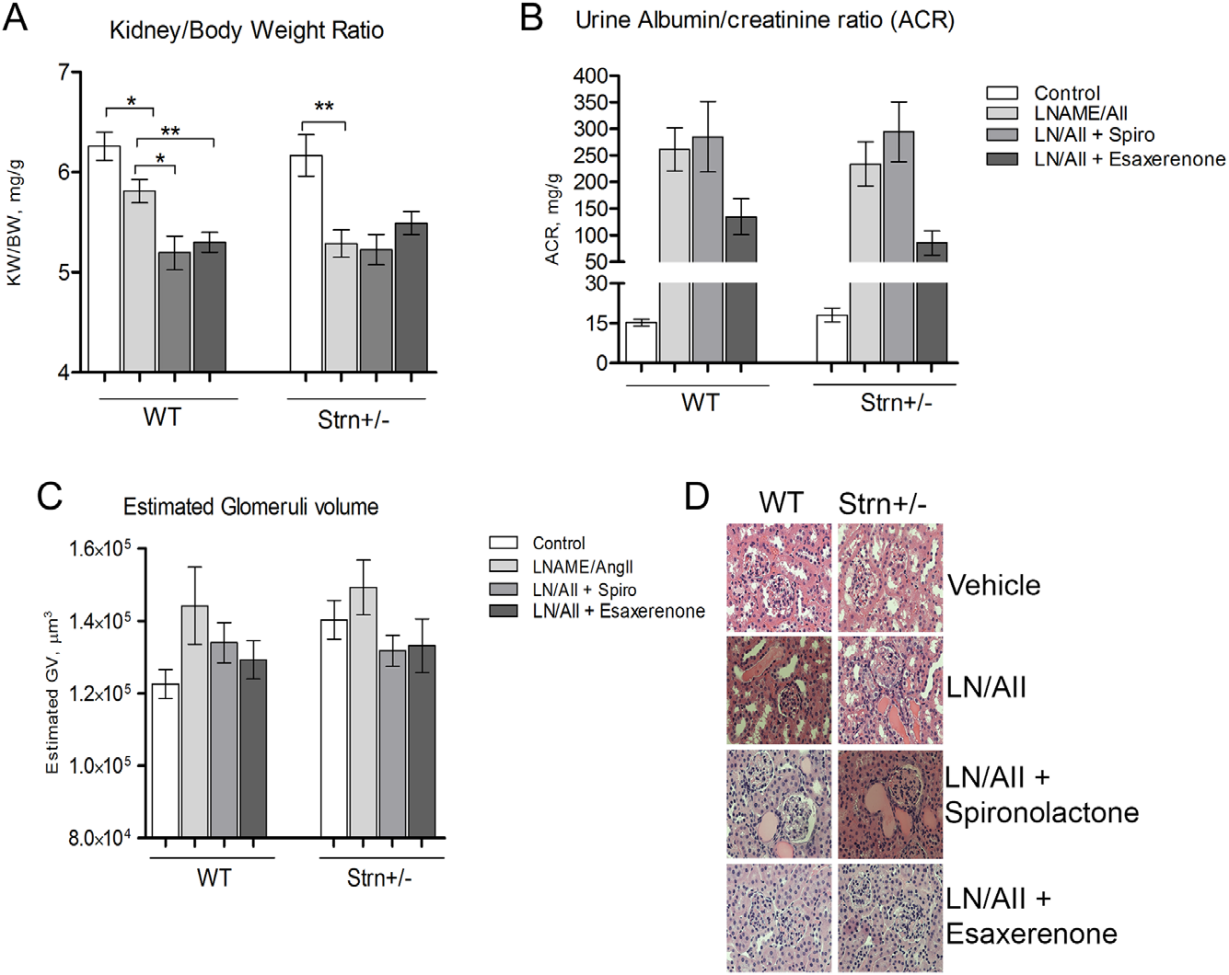
The effect of L-NAME/Ang II on renal injury. (A) Whole kidney weight to body ratio after 11 days of treatment. (B) Urine albumin/creatinine ratio was measured using DCA systems microalbumin/creatinine regent kit. (C) Periodic acid-Schiff (PAS) stained tissues were used to assess glomerular volume. (D) Representative glomeruli section H&E Staining, magnification 600×. Data presented as mean ± s.e.m., *n* = 6–8 mice/group. Data presented as mean ± s.e.m., *n* = 6–8 mice/group. Statistical analysis, * = *P* < 0.05, ** = *P* < 0.01 one-way ANOVA analysis comparing treatment groups within each genotype.

L-NAME/AngII treatment caused kidney atrophy in both the WT and Strn^+/–^ mice, as determined by the kidney to body weight (BW) ratio (Fig. 8A). The kidney atrophy is not prevented with spironolactone in either the WT or Strn^+/–^ mice. In both WT and Strn^+/–^ mice, esaxerenone treatment partially prevented the L-NAME/AngII-induced kidney atrophy; this effect was significantly different from that of spironolactone (WT: 4.8 ± 0.2 vs 5.4 ± 0.1, *P* < 0.05; Strn^+/–^4.4 ± 0.2 vs 5.1 ± 0.1, *P* < 0.05).

Microalbuminuria significantly increased by L-NAME/AngII treatment and similarly in the WT and Strn^+/–^ mice (Fig. 8B). Spironolactone is not able to prevent microalbuminuria in either the WT or Strn^+/–^ mice. In both WT and Strn^+/–^ mice, esaxerenone reduced the microalbuminuria to levels that were no longer significantly different from the vehicle treatment group. (Fig. 8B). Glomeruli volume was also increased in L-NAME/AngII-treated mice, but not significantly (Fig. 8C). Protein casts are present in the L-NAME/AngII-treated mice of both genotypes and in the spironolactone-treated mice but not present in the esaxerenone treatment group (Fig. 8D).

#### Striatin deficiency did not alter the response of the RAAS to L-NAME/AngII

Exogenous L-NAME and angiotensin II administration, in combination with a high sodium diet, suppresses the activity of the endogenous renin-angiotensin-aldosterone system (RAAS), as reflected in a significant decrease in plasma renin activity (PRA) levels (Fig. 6B). This physiological response was not modified by a reduction in striatin levels. Plasma aldosterone levels are markedly increased in the L-NAME/AngII-treated mice, both WT and Strn+/^–^ (Fig. 6A). MR blockade in the two genetic groups was similar. These data suggest that the reduction of striatin levels does not modify either the aldosterone or PRA levels in this model of injury. RAAS signaling. The renin-angiotensin-aldosterone system. Plasma renin activity is significantly decreased in response to L-NAME/AngII treatment.

## Discussion

We hypothesized that reducing the activity of aldosterone/MR’s non-genomic pathway would enhance cardiovascular and renal damage. To test this hypothesis, we used the Strn^+/–^ mouse since we have documented that striatin is a critical intermediary required to activate MR’s non-genomic pathway. Our hypothesis was proven correct, in part. For damage, likely secondary to increased volume expansion, for example, with aldosterone infusion, the Strn^+/^^–^ mouse had more damage than its WT littermate. Specifically, sodium-sensitive BP, glomerular volume and microalbuminuria were greater. However, striatin deficiency did not affect indices related to inflammation in the heart or the kidney in the aldosterone infusion model. Furthermore, in damage induced by a non-volume mediated mechanism, for example, L-NAME/AngII, striatin deficiency had no effect on the extent of damage, as assessed by any of the indices used. In general, blockade of the MR with either a steroid or non-steroid based MRA produced a similar reversal of the adverse effects, thereby documenting that in these models, the adverse effects are MR mediated.

Aldosterone plays an important role in a variety of physiological (e.g., BP and sodium, potassium and volume homeostasis) and pathophysiological (e.g., hypertension, cardiac and renal inflammation, and fibrosis, vascular dysregulation) conditions. It had been assumed that aldosterone’s pathophysiologic effects were an abnormal extension of its physiologic effects on epithelial cells and thereby mediated by an abnormality in volume homeostasis. For example, first, in both animal and human (primary aldosteronism) models of excess aldosterone production, minimal or no-damage occurred if sodium intake is severely restricted (Funder 2017). Second, in animal models, it is difficult to induce damage unless a kidney is removed (Sontia *et al.* 2008). Third, genetically or pharmacologically developed animal models of excessive MR activation required a liberal to very high sodium intake to induce damage. Fourth, sodium-sensitive BP was almost invariably a precursor of aldosterone-induced cardio-renal damage. However, recent studies have suggested that aldosterone-mediated cardiovascular/renal damage may not always require apparent increased epithelial cell activation. For example, activation of non-epithelial cells in the vasculature may also contribute, and likely initiate, inflammation, and fibrosis of heart, vessel, and kidney (Funder 2017).

The most convincing evidence for the non-epithelial cell effect comes from studies of heart failure and animal models where the endothelial function is impaired (Liu *et al.* 2003, Pitt *et al.* 2003). For example, in the L-NAME/AngII animal model, substantial cardio-renal damage is induced that can be prevented by adrenalectomy, salt restriction, or giving an MRA. Further, giving aldosterone to an adrenalectomized rat restores the damage (Rocha *et al.* 2000, Shibata *et al.* 2007). However, even though the damage is reduced or prevented by treatment, BP, and other indices of changes in volume status are not. In humans with heart failure or renal impairment, MR blockade reduces cardiovascular and renal damage without lowering BP or changing volume status except as would be anticipated by an improved cardiac status (Epstein *et al.* 2006). Thus, the currently available data suggest that there are two major mechanisms involved in mediating aldosterone-induced cardiovascular/renal damage: volume initiated (epithelial cell effect) and inflammation initiated (e.g., endothelial, inflammatory, immune cell effects). It also is likely that under some circumstances, both mechanisms may substantially contribute to the pathophysiology. The results of the current study suggest that when aldosterone is infused on a liberal salt diet, the damage induced is primarily epithelial cell (renal) driven. Salt sensitivity of BP, renal glomerular size and microalbuminuria were increased. However, histologic or molecular evidence for increased inflammation or fibrosis in the heart and kidney was minimal.

Furthermore, the results in the Strn^+/^^–^ mouse suggest that striatin’s role is to reduce volume-initiated damage as striatin deficiency increases salt-sensitive BP, glomerular size, and microalbuminuria, but did not affect histology or molecular markers of inflammation. This conclusion was reinforced by the results in the L-NAME/AngII model. As noted above, and observed in this study, the damage was likely mediated via endogenous aldosterone-activation of non-epithelial cells. As in the aldosterone-infused model, substantial injury in the heart and kidney was not modified in the mice with reduced striatin levels.

In addition to variations in the cell type targeted by aldosterone to induced cardiovascular and renal damage, the pathways used when MR is activated differ between classical genomic and rapid non-genomic. While the classical effect of MR activation leading to an aldosterone/MR complex that acts as a nuclear transcription factor has been known for more than 40 years, the concept of a rapid non-genomic effect was first proposed in the last decade of the past century (Wehling *et al.* 1992). Initially, it was thought to involved only non-epithelial cells and primarily involved in regulating intracellular ion concentrations, specifically the sodium/proton exchanger. However, more recent studies suggest that the activity of this pathway modulates epithelial cell function and modifies a variety of intracellular processes beyond intracellular ion content (Baudrand *et al.* 2014). The steroid non-genomic pathway is not limited to aldosterone, as other steroid receptors have been documented to activate both genomic and non-genomic pathways (Hammes & Levin 2007). Furthermore, multiple components of non-genomic aldosterone/MR signaling have been identified and demonstrated to effect classical MR signaling by either enhancing or repressing transactivation of MR genomic targets (Ruhs *et al.* 2017). Thus, like peptide receptors, after acutely modifying a second messenger’s level, for example, phosphorylating Akt, the cascade of downstream events can lead to changes in gene transcription. For example, we and others have shown that MR and striatin interact via caveolin-1 and that loss of caveolin-1 is also associated with adverse aldosterone/MR outcomes (Pojoga *et al.* 2010). Striatin’s function as a scaffold protein could be a key mediator to how multiple co-regulators can modulate MR’s transcriptional activity. We would propose that loss of striatin prevents a potential loss of repression signaling of MR’s transcriptional activation and therefore also upregulation of genomic targets, inappropriately. MR’s ability to balance physiology versus pathophysiologic signaling could be regulated by co-regulators such as striatin. However, despite nearly three decades of research related to steroids’ non-genomic pathways, uncertainty exists as to their unique mechanisms and a steroids’ interaction between its genomic and non-genomic pathways.

Several studies have documented striatin’s critical role in mediating aldosterone’s non-genomic mechanism of action. (1) *In vitro* in mouse and human endothelial cell systems, aldosterone increases striatin mRNA and protein levels (Pojoga *et al.* 2012). (2) Dietary salt restriction increases striatin protein levels in mouse hearts (Pojoga *et al.* 2012). (3) Intraperitoneal administration of aldosterone increases striatin protein levels in mouse hearts (Pojoga *et al.* 2012). (4) *In vitro* in both human and mouse endothelial cell systems, aldosterone increases the rapid phosphorylation of extracellular signal-regulated protein kinases 1/2 (ERK 1/2) and alpha serine/threonine-protein kinase (Akt) (Coutinho *et al.* 2014, Garza *et al.* 2015*b*). (5) Reducing striatin levels with siRNA prevents the rise in pERK and pAkt levels but does not modify aldosterone’s genomic effects (Coutinho *et al.* 2014, Garza *et al.* 2015*b*). (6) MR co-precipitates with striatin in vascular tissue (Pojoga *et al.* 2012). Thus, reducing striatin levels via genomic mutation will inhibit aldosterone’s non-genomic pathway. Based on these data, the increase in volume mediated aldosterone damage observed in the striatin heterozygote knockout mouse suggests that aldosterone’s non-genomic pathway protects against gnomically mediated damage. This is like what has been demonstrated for the estrogen receptor. In a rodent model of vascular injury produced by carotid artery wire damage, estrogen administration is protective. However, this estrogen-mediated protection was abolished in a transgenic mouse model, where the estrogen receptor’s non-genomic effect was prevented by overexpression of a protein that blocked the receptor’s binding to striatin (Coutinho *et al.* 2014). Whether similar effects are mediated by the non-genomic pathway of other steroid receptors is unknown. However, the presently available data strongly suggest that striatin is critically involved in mediating the activation of a steroid’s non-genomic pathway.

Aldosterone did not induce hypertension in our WT mice, which is not entirely unexpected and reported previously (Sontia *et al.* 2008). Aldosterone-induced hypertension is primarily observed under conditions such as (1) uninephrectomy (Sontia *et al.* 2008); (2) longer duration of aldosterone or increased concentration; or (3) rodent model with kidney dysfunction. Therefore, the significant rise in BP in our Strn^+/^^–^ mice demonstrates the importance of striatin (and the non-genomic pathway) in limiting aldosterone’s adverse effect on BP. It appears that this non-genomic protection is not extended to the vascular/inflammation/immune effects of aldosterone as these adverse effects in the heart and kidney (histologically and molecularly) did not differ between the Strn^+/^^–^ and WT mice in either model used whether the damage was minimal (aldosterone infused) or substantial (L-NAME/AngII).

What is the clinical relevance of these results? Because striatin levels substantially influence the degree of volume (epithelial) mediated damage induced by aldosterone, changes in its levels could, in part, modify aldosterone’s adverse cardiovascular effects without modifying its effects on potassium homeostasis. For example, the increase in aldosterone mediated cardiovascular damage may not just be secondary to an increase in its levels but also could be secondary to a decrease in striatin levels. Alternatively, the development of agents that can increase striatin levels may be beneficial in reducing aldosterone’s adverse effects.

There are several limitations to our findings. First, could the effects observed be secondary to a non-MR mediated aldosterone effect? This is unlikely since two MR antagonists were used in this study: two classical steroid-based molecules – eplerenone and spironolactone – and a pyrrole derivative, selective and highly potent MR antagonist with long-lasting activity, esaxerenone (Arai *et al.* 2015). The molecular structure of MR antagonists can impact potency and selectivity and to assess the specificity of our findings were related to activation of the MR, we used three MRA’s – eplerenone and spironolactone (steroid based) and esaxerenone (non-steroid based). Each steroidal MRA selected for each study has previously been shown to block injury in a similar model (Rocha *et al.* 2002, Oestreicher *et al.* 2003). Esaxerenone has a different pharmacological profile than spironolactone and eplerenone (Arai *et al.* 2015). While not all adverse effects were equally reversed by both antagonists, there was a consistent overall reduction with MR blockade, thus supporting the hypothesis that these adverse effects were MR mediated, not off target effects of the antagonist. Some of the differences likely are related to dose-response differences that could have been addressed by using a dose titration paradigm rather than the single-dose one used. Second, some of the effects seen could be due to the liberal salt intake itself rather than aldosterone since no studies were performed on low salt intake. Again, the responses to the MR antagonists makes this unlikely. Third, while it appears as if striatin deficiency did not modify aldosterone’s effects on non-epithelial cells in contrast to epithelial cells, our study designs did not allow for a definitive answer. Yet, similar findings with the two different protocols support this hypothesis. Finally, we do not know whether or not the responses would be the same in females versus males, particularly since it has been previously shown that striatin deficiency reduces estrogen’s cardiovascular protective effect (Bernelot Moens *et al.* 2012).

In conclusion, striatin is a major mediator of aldosterone’s non-genomic pathway. The reduced striatin level in the striatin heterozygote knock-out mouse is associated with increased aldosterone-induced cardiovascular/renal damage, suggesting that the activity of its non-genomic pathway ameliorates adverse aldosterone effects. However, the relationship between striatin levels and aldosterone’s adverse effects was selective and only appeared to modify aldosterone’s epithelial but not its non-epithelial-mediated actions.

## Declaration of interest

The authors declare that there is no conflict of interest that could be perceived as prejudicing the impartiality of the research reported.

## Funding

This work was supported by the NHLBI Grants R01HL11476 (G H W), R01HL104032 (L H P), R01HL096518 (J R R), a NIH T32 training grant T32HL007609-27 (D B, S R), Chilean National Science and Technology Research Fund (FONDECYT) 1130427 (R B) and Grant-in-aid Daiichi Sankyo Company Limited, Japan who also supplied esaxerenone and eplerenone for this study.
